# Effects of velocity loss with blood flow restriction in full squat on strength gains, neuromuscular adaptations, and muscle hypertrophy

**DOI:** 10.5114/biolsport.2025.151657

**Published:** 2025-08-05

**Authors:** Juan Sánchez-Valdepeñas, Luis Rodiles-Guerrero, Pedro Jesús Cornejo-Daza, Jose Antonio Paez-Maldonado, Clara Cano-Castillo, Beatriz Bachero-Mena, Miguel Sánchez-Moreno, Juan José González-Badillo, Eduardo Saez de Villarreal, Fernando Pareja-Blanco

**Affiliations:** 1 Physical Education and Sports Department, Cardenal Spínola CEU Andalucía University, Bormujos, Sevilla, Spain; 2 Science-Based Training Research Group, Physical Performance and Sports Research Center, Universidad Pablo de Olavide, Seville, Spain; 3 Department of Human Movement and Sport Performance, University of Seville, Spain; 4 Faculty of Sports Sciences, Department of Sports and Computer Sciences, Universidad Pablo de Olavide, Seville, Spain; 5 University of Osuna (Centre attached to the University of Seville), Osuna, Spain; 6 Department of Physical Education and Sports, University of Seville, Seville, Spain

**Keywords:** Velocity-based training, Full squat, Muscle size, Strength, Gains, Jump performance

## Abstract

To analyse the effects of four full squat (SQ) training programmes with different velocity loss (VL) thresholds (0%, 10%, 20%, and 40%) with blood flow restriction (BFR) implementation on muscle size, lower limb strength, and neuromuscular adaptations. Forty-six strength-trained men carried out an 8-week (16 sessions) SQ training programme with BFR that differed in the VL attained within the set: BFR 0% VL (BFR0, n = 11), BFR 10% VL (BFR10, n = 11), BFR 20% VL (BFR20, n = 11), and BFR 40% VL (BFR40, n = 13). The same inter-set recovery (2 minutes), sets (3), intensity (from 55% to 70% 1RM), and level of BFR (50% of arterial occlusion pressure) were established for all groups. Before and after the training intervention, the following tests were carried out: 1) vastus lateralis muscle size; 2) countermovement jump; 3) maximal isometric SQ test; 4) progressive loading SQ test; and 5) fatigue SQ test. Muscle hypertrophy increased as the VL increased (“group × time” interaction: p = 0.013). Only BFR20 significantly improved force production at various time intervals (“group × time” interactions: p ≤ 0.05). Moreover, effect sizes suggest that low-to-moderate VL thresholds maximize the improvements in SQ strength against different loads (BFR0: 0.47–1.75; BFR10: 0.61–1.96; BFR20: 0.71–2.18; BFR40: 0.38–1.53). In BFR contexts, low-to-moderate VL thresholds should be prescribed to optimize leg strength performance. Extremely low VL (i.e., 0%) seemed insufficient to maximize strength gains, while higher VL thresholds are more effective for promoting muscle hypertrophy but may somewhat compromise strength improvements.

## INTRODUCTION

Interest in resistance exercise under partial blood flow restriction (BFR) has thoroughly increased owing to the promising long-term effects on muscular development across various populations [1–3]. Notably, BFR during resistance exercise (BFR-RE) enhances strength and hypertrophy gains compared to free flow resistance exercise (FF-RE) with low relative loads [2]. Researchers and practitioners usually implement BFR-RE against low loads (20–50% of 1-repetition maximum (1RM)), with volume protocols such as 4 sets of 30/15/15/15 repetitions or sets performed to failure [4, 5]. Following this approach, a few studies have investigated the effects of different training volumes within a BFR context. Bjornsen et al. [6] compared failure sets vs. non-failure protocols (30/15/15/15) at 20% 1RM across 14 sessions, observing significant increases in muscle strength, myonuclei content, and muscle hypertrophy with both approaches. Similarly, Sieljacks et al. [7] demonstrated that the non-failure protocol (performing 75% of repetitions relative to failure) produced equivalent increases in quadriceps size, local endurance, and muscle strength as the same protocol carried out to muscle failure. These findings suggest that inducing high fatigue levels may not be necessary to maximize strength and hypertrophic adaptations with BFR. While BFR can elicit substantial muscle damage [8, 9] and considerable myocellular stress [10, 11], moderate-fatigue protocols may balance efficacy with reduced recovery demands, supporting their broader application in training regimens.

Velocity loss (VL) has been proposed as a key metric for monitoring intra-set fatigue due to its association with metabolic responses and performance impairments across varying resistance training configurations [12]. Moreover, this parameter has also shown a close relationship with the percentage of repetitions performed within the set [13, 14], providing valuable insight into the level of effort attained. Regarding long-term adaptations, VL is considered a critical variable in determining the adaptations to resistance training programmes. When volume is prescribed via VL, a low to moderate intra-set level of effort is enough to elicit substantial gains in strength and specific athletic performance tasks and neuromuscular adaptations [15–18]. Notably, the dose-response relationship between VL thresholds and performance adaptations followed an inverted Ushape curve [16–18]. This means that a certain VL is required to maximize strength gains. However, once this optimal VL is reached within the set, performing additional repetitions may not only be superfluous but could also be detrimental to strength gains. In contrast, higher VL thresholds are generally required to maximize muscle hypertrophy [15, 16, 18]. However, this comes with trade-offs: for instance, a high VL (e.g., 40%) in full squat (SQ) decreased the myosin heavy chain IIX percentage in the vastus lateralis muscle [15]. This could adversely impact neuromuscular adaptations, especially when high-velocity actions or force production in a short time is prioritized. Thus, tailoring the VL within the set allows practitioners to align training adaptations with specific training goals.

Although VL has been proposed as a valuable tool for prescribing real-time fatigue and level of effort within a set, the long-term effects of implementing different VL thresholds in the BFR context remain unexplored. Therefore, more insight is necessary to establish how much exertion would be enough to maximize strength gains and muscle size when implementing BFR. This study aimed to analyse the effects of different VL thresholds during SQ training with BFR on strength gains, neuromuscular adaptations, and muscle hypertrophy.

## MATERIALS AND METHODS

### Experimental Design

A study with a longitudinal research design was carried out to analyse the effects of four resistance training interventions based on the SQ exercise with BFR, differing in the VL threshold achieved within the set: 0% (BFR0); 10% (BFR10); 20% (BFR20) and 40% (BFR40). Participants completed 16 sessions during 8 weeks twice a week (at least 48 hours apart). All training interventions were designed with the same relative load (from 55% to 70% of 1RM), number of sets (3), and inter-set recovery (2 minutes). Regarding BFR implementation, 50% of the arterial occlusion pressure (AOP) was adjusted and maintained during the whole training session, including inter-set recovery time but excluding the warm-up. Two testing sessions were conducted, 72 and 96 hours before and after the training intervention. Anthropometric measurements and the anatomical cross-sectional area (ACSA) of the vastus lateralis muscle were obtained in the first testing session. During the second testing session, a battery of tests was performed in the following order: 1) countermovement jump (CMJ); and the following tests in the SQ exercise: 2) maximal voluntary isometric contraction (MVIC); 3) progressive loading test; and 4) fatigue test. Participants were encouraged to exert maximum effort during the test and training sessions. All measurements were performed in a research laboratory under the direct supervision of trained researchers at the same time of day (± 1 h) for each subject with the same environmental conditions (20°C and 60% humidity).

### Subjects

Fifty-six strength-trained men (mean ± SD: height = 1.78 ± 0.05 m; age = 22.7 ± 4.4 years; body mass = 75.2 ± 7.7 kg; 1RM/body mass = 1.37 ± 0.29) with at least one year of resistance training background volunteered to take part in this study. Before the beginning of the study, participants were informed about the potential risk of the intervention process and, after that, signed a written informed consent form. Subjects were randomly assigned according to their 1RM to one of four groups. Ten subjects dropped out due to reasons unrelated to the study. The remaining sample comprised 46 subjects: BFR0 (n = 11), BFR10 (n = 11), BFR20 (n = 11), and BFR40 (n = 13). Subjects did not present physical limitations or health problems that could disturb their participation in the intervention. To participate, subjects had to meet the requirements regarding resistance training experience and be able to perform the SQ exercise correctly. The present study was approved by the Research Ethics Committee of Universitarios Virgen Macarena-Virgen del Rocío (Reference: 1547-N-19) in accordance with the Declaration of Helsinki.

### Testing Procedures Ultrasound Echography

Measurements of ACSA were carried out using B-mode ultrasonography (MyLab 25, Esaote Biomedica, Italy). Before the measurements, participants remained lying down for 15 minutes. After that, with the knees flexed at 150°, panoramic images of the vastus lateralis were measured at 40% (distal; ACSA_40_), 57.5% (medial; ACSA_57.5_), and 75% (proximal; ACSA_75_) of the femur length, defined as the distance between the bottom of the lateral condyle and the top of the greater trochanter [19]. Assessments were carefully taken from the medial to lateral side at a constant velocity with the ultrasound linear array probe perpendicular to the skin’s surface. To ensure the suitable direction of the probe, a couple of adhesive gaskets were located close to both sides of the transverse path. The expert researcher applied the minimum pressure during the measurements to avoid deformations in the evaluated tissue. To ensure consistency in measurement places at pre- and post-training testing, a transparent acetate with identifiable infiltrations of fatty and connective tissues as landmarks was recorded per each subject. Three images were obtained at each thigh length (i.e., 75%, 57.5%, and 40%) and digitally analysed (ImageJ 1.51j8, NIH) by the same expert researcher. Muscle volume (cm^3^) was estimated using the cubic spline interpolation method [20] and specialized software (OriginPro 8.5, OriginLab, USA) as proposed by Cornejo-Daza et al. [21]. The average value of two images per ACSA point was selected for further analysis. If the coefficient of variation (CV) was greater than 5%, a third image was included in the subsequent analysis. Our laboratory’s test-retest CV for the vastus lateralis ACSA is 3.8% [16].

### Countermovement Jump Test

Jump height was measured using an infrared timing system (OptojumpNext, Microgate, Bolzano, Italy). Participants performed five maximal CMJs with a 20 s recovery between attempts. Both hands should rest on the hips while performing a downward movement followed by a vertical jump of maximum effort. Subjects were instructed to keep their knees straight during the flight phase and to land in an upright position. The best and the worst attempts were removed, and the mean of the 3 remaining values were used for further analysis. The warm-up consisted of two sets of 10 body mass SQs, 5 CMJs with progressively increasing intensity and 3 close-tomaximal effort CMJs.

### Maximal Voluntary Isometric Squat Test

Subjects performed an MVIC test on a Smith Machine (Multipower Fitness Line, Peroga, Murcia, Spain) with a precision scale placed at both sides before standardizing the initial position with knees flexed at 90º in SQ. Participants were instructed to push the bar as fast and hard as possible for ~ 5 seconds per trial. Two attempts with a 1-minute recovery were performed. Force data were collected with a force plate at a sampling rate of 1000 Hz and analysed with specific software (both T-Force System, Ergotech, Murcia, Spain). The following outcomes were considered for further analysis: maximal isometric force (MIF); maximal rate of force development (RFDmax), which was established as the maximum slope in the force-time curve in 20 ms time intervals; and c) the average tangential slope of the force-time curve achieved over different time intervals (50, 100, 200, and 400 ms from the force production onset; RFD_0–50_, RFD_0–100_, RFD_0–200_, and RFD_0–400_, respectively). The average value of the two attempts for each variable was used for further analysis. The onset of the force signal was established when the signal increased above 2 standard deviations (SDs) from the baseline signal.

### Progressive Full-Squat Loading Test

This test was executed on a Smith machine (Multipower Fitness Line, Peroga, Murcia, Spain) with no counterweight mechanism, and a linear velocity transducer (T-Force System Ergotech, Murcia, Spain) was used to record all repetitions. Participants started from the upright position with the hips and knees fully extended, the barbell resting across the back at the level of the acromion, and the stance approximately shoulder-width apart. The eccentric phase was instructed to be performed in a continuous motion at a controlled mean velocity (~0.50–0.65 m · s^-1^), as low as possible for each subject, at least until the top of the thighs was below the horizontal plane (~35–40° knee flexion). After that, the concentric phase was executed without a pause at maximal velocity. The initial common absolute load was 20 kg and progressively increased in steps of 10 kg until the attained mean propulsive velocity (MPV) was ≤ 0.5 m · s^-1^. Nevertheless, the load was increased with smaller increments (5 down to 2.5 kg) for better adjustment when velocity was close to 0.5 m · s^-1^. Three repetitions were performed for a light load (≥ 1.00 m · s^-1^), two for a moderate load (1.00–0.80 m · s^-1^), and one for the heaviest loads (≤ 0.80 m · s^-1^). Inter-set recoveries ranged from 3 minutes (light) to 5 minutes (heavy loads). An identical absolute load progression was carried out at the post-training test. The best repetition against each load, following the criteria of the fastest MPV, was considered for further analysis. The 1RM was determined from the individual lineal load-velocity relationship as the load that could be lifted at 0.32 m · s^-1^ [22]. In addition to the 1RM load, the following variables derived were analysed: a) AV: average MPV attained against absolute loads common to pre- and post-test; b) AV ≥ 1: average MPV attained against absolute loads common to pre- and posttraining that were moved equal or faster than 1 m · s^–1^ and c) AV < 1: average MPV attained against absolute loads common to pre- and post-training that were moved slower than 1 m · s^–1^. The specific warm-up consisted of 6 SQ repetitions with 20 kg. The velocity outcome corresponds to the propulsive phase of each repetition, defined as that phase of the concentric action in which the measured acceleration is greater than gravity acceleration (-9.81 m · s^-2^) [23].

### Fatigue Test

Participants carried out this test with the same absolute load (kg) corresponding to 70% 1RM load at pre-training 5 min after the incremental loading test. Subjects were instructed to perform as many repetitions as possible until the MPV was less than 0.50 m · s^-1^. Devices and technique tips were similar to those described in the “Progressive Full-Squat Loading Test” section. The number of repetitions completed during this test (MNR) was considered for further analyses.

### Arterial Occlusion Pressure

The AOP was determined to individualize the BFR level during the training intervention. Before the measurements, participants remained seated on the edge of a chair with 90° hip and knee flexion for 10 minutes. A couple of cuffs (12 cm wide × 86 cm length; VBM20-54-528, Medizintechnik, GmbH, Germany) were placed on each leg proximal to the groin part of the thigh. The pulse was detected over the posterior tibial artery using a hand-held Doppler probe (Dopplex D900, Huntleigh Healthcare Ltd., UK) with an inclination of 60º degrees. Then, the cuff was inflated to 70 mmHg using a manual inflator (VBM20-18-601-VBM20-18-602, Medizintechnik, GmbH, Germany), and pressure was incrementally increased until the auscultatory pulse was no longer present, in steps of 10 to 10 mmHg and 5 to 5 mmHg when the pulse was not so long [24]. The lowest cuff pressure at which the pulse was not recorded was established as the AOP. This procedure was repeated for both legs twice, with 5 minutes of rest between attempts for each leg. The mean value of the 2 measurements of the AOP was used. This procedure was repeated at a mid-point of the resistance training intervention before starting the fifth week, avoiding susceptible variations. Mean AOP values for both assessments were 235.3 ± 27.5 and 235.9 ± 22.7 mmHg for the right leg and 234.05 ± 22.9 and 235.7 ± 21.7 mmHg for the left leg, without significant differences between them.

### Resistance Training Programme

Participants completed an 8-week training programme, 2 sessions per week, using the SQ exercise with BFR implementation. The same relative intensity (55–70% 1RM), number of sets (3), inter-set recoveries (2 minutes), and level of BFR (50% AOP) were prescribed for all training groups. The training interventions differed in the VL threshold allowed in each set (BFR0 vs. BFR10 vs. BFR20 vs. BFR40). Hence, groups stopped the set when the corresponding target VL threshold was achieved. Notably, BFR0 only performed one repetition per set, reaching the minimum amount of possible induced fatigue. All repetitions were recorded using a linear velocity transducer (T-Force System, Ergotech, Murcia, Spain). The following standardized warm-up was carried out before each training session: 5 minutes of jogging at a self-selected leisurely pace, two sets of 10 SQs with their body mass, and a set of 6 repetitions with 20 kg on a Smith machine, followed by two sets of 6 and 4 SQ repetitions with 40% and 50% 1RM, respectively, for sessions 1–8, in which the training load was 55–60% 1RM. A set of 3 SQ repetitions with 60% 1RM was added for sessions 9–16, in which the training load was 65–70% 1RM. A 3-minute rest between the warm-up sets was always used. Performance (i.e. MPV and absolute load) against the 50% 1RM load during the warm-up in every training session was analysed to evaluate the evolution of performance throughout the training programme. The values derived from individual load-velocity relationships were employed to prescribe the training load in each training session, including the warm-up. Therefore, individual loads were adjusted for each training session to ensure that the corresponding MPV matched (± 0.03 m · s^-1^) the prescribed %1RM. Previous research has shown that 0.03 m · s^-1^ is the smallest detectable change in MPV for the SQ exercise [25]. The following variables were calculated from the training programme conducted by each group: fastest MPV – average MPV of the fastest repetitions performed in each session; slowest MPV – average MPV of the slowest repetitions performed in each session; MPV all reps – average MPV attained during the entire training programme; mean VL – average VL attained during the training programme; total rep – total repetitions performed during the training programme; reps per set in all sessions – average repetitions performed in each set; reps per set with a given %1RM – average repetitions performed in each set per relative intensity (i.e., 55%, 60%, 65%, and 70% 1RM).

### Statistical Analysis

Data are presented as mean ± SD. Data from the intraclass correlation coefficient with 95% confidence intervals, using the one-way random effects model, and CV are reported regarding reliability outcomes. Shapiro-Wilk and Levene’s tests were conducted to ensure the data normality and homoscedasticity, respectively. Data were analysed using a 4 × 2 factorial ANOVA with Bonferroni’s post hoc comparisons using one between-group factor (BFR0 vs. BFR10 vs. BFR20 vs. BFR40) and one within-group factor (pre- vs. post-training). The characteristics of the training programmes conducted were compared using a one-way ANOVA. Statistical significance was set at the level p ≤ 0.05. Effect size (ES) values were calculated using Hedge’s g [26] with a purpose-built spreadsheet. All statistical analyses were conducted using SPSS Statistics software version 20.0 (IBM Corp.) and figures were designed with GraphPad Prism 8.0.0 (GraphPad Software, San Diego, CA, USA).

## RESULTS

Compliance was 100% for all participants. No significant differences between groups were found during pre-training (from p = 0.54 to p = 1.00) for any of the parameters assessed. Table 1 shows the test-retest reliability for different variables analysed.

**TABLE 1:** The reliability values of the different tests conducted.

Parameter	ICC (95% CI)	CV %
CMJ	1.00 (0.99–1.00)	1.7 (1.3–2.3)
MIF	0.97 (0.94–0.98)	5.9 (4.4–7.9)
RFDmax	0.86 (0.74–0.93)	16.1 (12.0–21.6)
RFD0–50	0.86 (0.73–0.93)	25.7 (19.1–34.6)
RFD_0–100_	0.88 (0.75–0.94)	21.5 (16.0–28.9)
RFD_0–200_	0.84 (0.70–0.92)	18.5 (13.8–24.8)
RFD_0–400_	0.82 (0.65–0.91)	15.0 (11.2–20.1)

ICC: intraclass correlation coefficient; CI: confidence interval; CV: coefficient of variation; CMJ: countermovement jump; MIF: maximal isometric force; RFDmax: maximal rate of force development;

RFD_0–50_: RFD in the first 50 ms; D) RFD_0–100_: RFD in the first 100 ms; E) RFD_0–200_: RFD in the first 200 ms; and F) RFD_0–400_: RFD in the first 400 ms.

### Training Programme

The main characteristics of the training programmes are displayed in Table 2. No significant differences between groups were observed for the fastest MPV (p > 0.05). The higher the VL, the lower was the MPV for all repetitions completed and the slowest MPV (all p < 0.05). Likewise, the greater the VL, the higher were the total repetitions and the repetitions per set completed (p < 0.05).

**TABLE 2:** Descriptive characteristics of the 8-week velocity-based squat training program with blood flow restriction performed by the four groups.

***Scheduled***	**Session 1**	**Session 2**	**Session 3**	**Session 4**	**Session 5**	**Session 6**	**Session 7**	**Session 8**

**Set × %1RM**	3 × 55	3 × 55	3 × 55	3 × 55	3 × 60	3 × 60	3 × 60	3 × 60
**Target Velocity (m · s^-1^)**	0.97 ± 0.07	0.97 ± 0.07	0.97 ± 0.07	0.97 ± 0.07	0.90 ± 0.07	0.90 ± 0.07	0.90 ± 0.07	0.90 ± 0.07

***Scheduled***	**Session 9**	**Session 10**	**Session 11**	**Session 12**	**Session 13**	**Session 14**	**Session 15**	**Session 16**

**Set × %1RM**	3 × 65	3 × 65	3 × 65	3 × 65	3 × 70	3 × 70	3 × 70	3 × 70
**Target Velocity (m · s^-1^)**	0.83 ± 0.06	0.83 ± 0.06	0.83 ± 0.06	0.83 ± 0.06	0.76 ± 0.05	0.76 ± 0.05	0.76 ± 0.05	0.76 ± 0.05



***Actually Performed***	**Fastest MPV (m · s^-1^)**	**Slowest MPV (m · s^-1^)**		**MPV all reps (m · s^-1^)**		**Mean VL (%)**	**Total Rep**	

BFR0	0.90 ± 0.04	0.84 ± 0.05 ^10 20 40^		0.87 ± 0.04 ^20 40^		0.0 ± 0.0 ^10 20 40^	48.0 ± 0.0 ^10 20 40^	
BFR10	0.89 ± 0.07	0.73 ± 0.07 ^20 40^		0.82 ± 0.07 ^20 40^		11.4 ± 0.7 ^20 40^	197.5 ± 42.1 ^20 40^	
BFR20	0.87 ± 0.07	0.62 ± 0.06 ^40^		0.75 ± 0.07 ^40^		21.1 ± 0.9 ^40^	285.7 ± 91.9 ^40^	
BFR40	0.85 ± 0.04	0.42 ± 0.03		0.64 ± 0.04		41.2 ± 1.3	384.8 ± 61.4	

***Actually Performed***	**Rep per set in all sessions**	**Rep per set with 55% 1RM**		**Rep per set with 60% 1RM**		**Rep per set with 65% 1RM**	**Rep per set with 70% 1RM**	

BFR0	1.0 ± 0.0 ^10 20 40^	1.0 ± 0.0 ^10 20 40^		1.0 ± 0.0 ^10 20 40^		1.0 ± 0.0 ^10 20 40^	1.0 ± 0.0 ^10 20 40^	
BFR10	4.1 ± 1.3 ^20 40^	5.1 ± 1.6 ^20 40^		4.3 ± 1.0 ^20 40^		3.8 ± 0.9 ^40^	3.3 ± 1.0 ^40^	
BFR20	6.1 ± 2.0 ^40^	8.2 ± 2.6 ^40^		7.3 ± 2.7 ^40^		4.8 ± 1.9 ^40^	3.9 ± 1.3 ^40^	
BFR40	8.0 ± 1.3	10.1 ± 2.2		8.9 ± 1.7		7.2 ± 1.5	5.9 ± 1.0	

Data are mean ± SD. BFR0: Group that trained with a velocity loss (VL) of 0% in each set (n = 11); BFR10: Group that trained with a VL of 10% in each set (n = 11); BFR20: Group that trained with a VL of 20% in each set (n = 11); BFR40: Group that trained with a VL of 40% in each set (n = 13); Fastest MPV: Average mean propulsive velocity (MPV) of the fastest repetitions performed in each session; Slowest MPV: Average MPV of the slowest repetitions performed in each session; MPV all reps: Average MPV attained during the entire training program; Mean VL: Average VL attained during the training program; Total rep: Total repetitions performed during the training program; Rep per set in all sessions: average repetitions performed in each set; Rep per set with a given %1RM: average repetitions performed in each set per relative intensity (i.e. 55, 60, 65 and 70%1RM). Statistically significant differences with BFR10 group: ^10^ p < 0.05. Statistically significant differences with BFR20 group: ^20^ p < 0.05. Statistically significant differences with BFR40 group: ^40^ p < 0.05.

### Muscle Hypertrophy

**FIG. 1:**
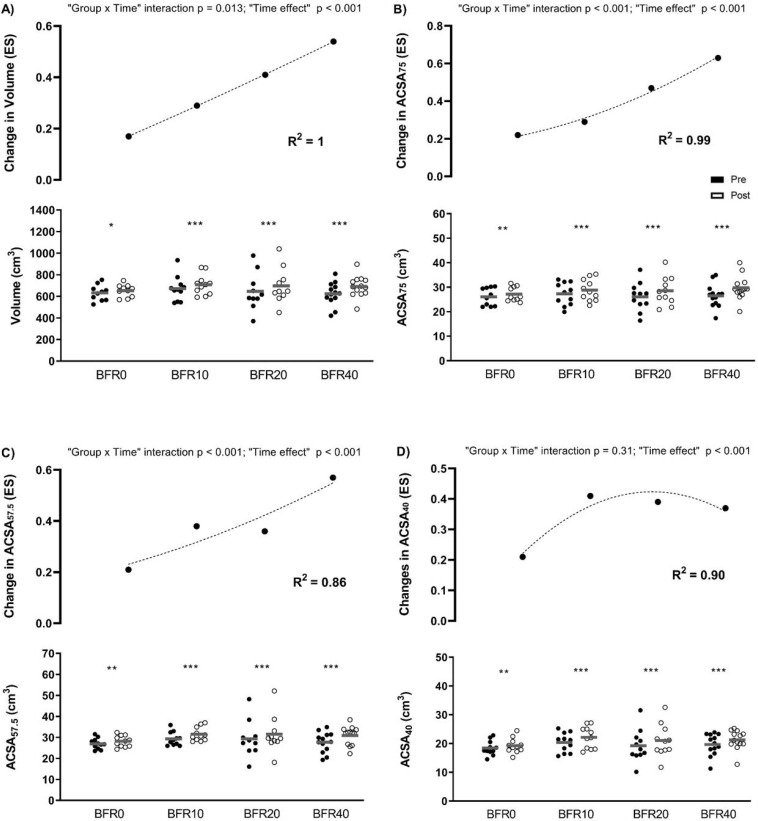
Changes in muscle size following full-squat blood flow restricted training programmes with different velocity loss (VL) thresholds. A) Volume: volume of the vastus lateralis (VLA) muscle; B) ACSA75: anatomical cross-sectional area (ACSA) of the VLA at 75% of the femur length; C) ACSA57.7: ACSA of the VLA at 57.5% of the femur length; D) ACSA40: ACSA of the VLA at 40% of the femur length; ES: effect size; BFR0: group training with a 0% VL; BFR10: group training with a 10% VL; BFR20: group training with a 20% VL; BFR40: group training with a 40% VL. Intra-group significant differences from pre- to post-intervention: * p ≤ 0.05, ** p ≤ 0.01, *** p ≤ 0.001.

Significant “group × time” interactions were observed for muscle volume, ACSA_75_, and ACSA_57.5_ (from p = 0.01 to p < 0.001). The higher the VL attained, the greater was the increase produced in these variables (Figure 1).

### Maximal Isometric Force and Force Production at Different Time Intervals

**FIG. 2:**
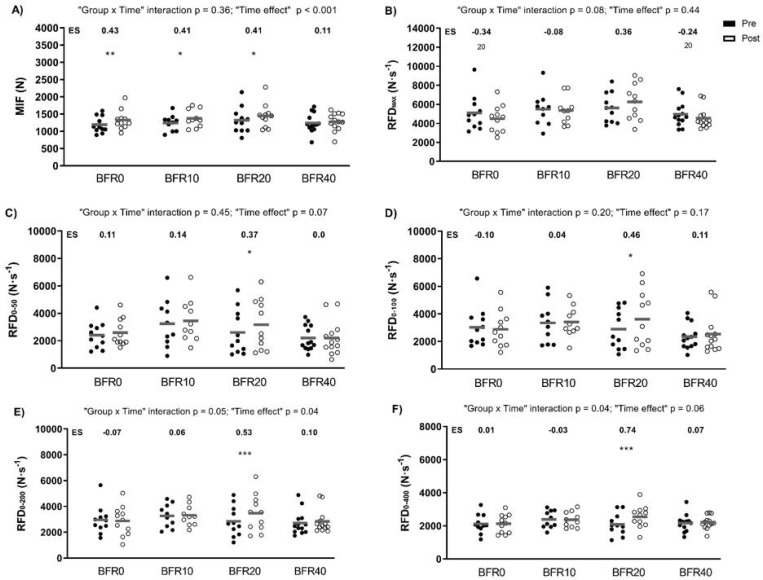
Changes in force production and rate of force development (RFD) following full-squat blood flow restricted training programmes with different velocity loss (VL) thresholds. A) MIF: maximal isometric force; B) RFDmax: maximal RFD; C) RFD0-50: RFD in the first 50 ms; D) RFD0-100: RFD in the first 100 ms; E) RFD0-200: RFD in the first 200 ms; and F) RFD0-400: RFD in the first 400 ms; ES: effect size; BFR0: group training with a 0% VL; BFR10: group training with a 10% VL; BFR20: group training with a 20% VL; BFR40: group training with a 40% VL. Intra-group significant differences from pre- to post-intervention: * p ≤ 0.05, ** p ≤ 0.01, *** p ≤ 0.001.

Figure 2 depicts the data derived from the MVIC test. Significant main “time” effects were found for MIF (p < 0.001) and RFD_0–200_ (p = 0.04). In all groups except BFR40, MIF increased significantly. Significant “group × time” interactions were observed for RFD_0–200_ and RFD_0–400_ (p = 0.05 and p = 0.04, respectively). Only BFR20 induced significant increases in RFD at different time intervals (from RFD_0–50_ to RFD_0–400_; p = 0.001–0.05).

### Vertical Jump Performance

**FIG. 3:**
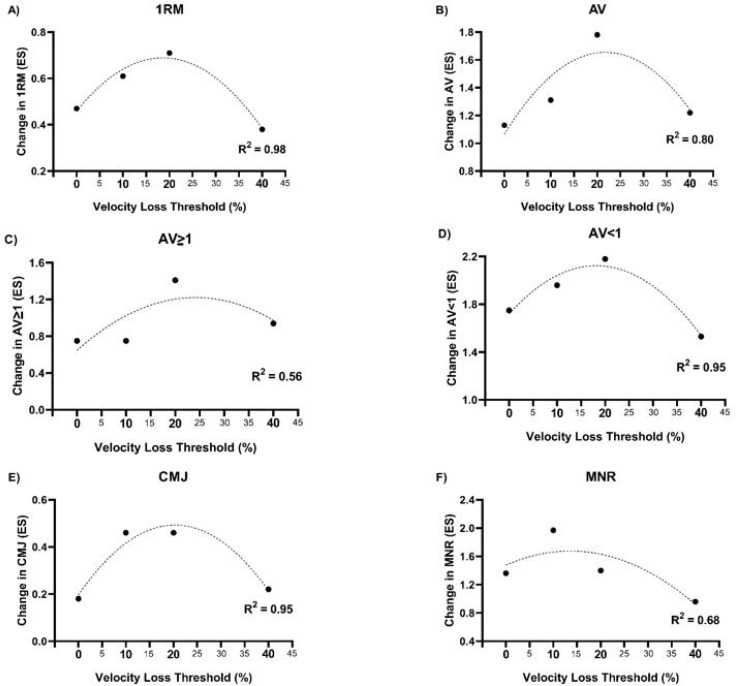
Relationship between velocity loss in the set and within-group effect size from pre- to post-training in: 1RM (A); average MPV attained against absolute loads common to pre-to post-training (AV) (B); average MPV attained against absolute loads common to pre-to post-training that were moved equal or faster than 1 m · s^-1^ at pre-training (AV ≥ 1) (C); average MPV attained against absolute loads common to pre- to post-training that were moved slower than 1 m · s^-1^ at pre-training (AV < 1) (D); countermovement jump (CMJ) height (E); and maximal number of repetition performed in the fatigue test (F).1

A significant main “time” effect (p < 0.001) and a non-significant “group × time” interaction (p = 0.06) were observed for CMJ height. The ES values showed an inverted-U response for the different groups: 0.18, 0.46, 0.46, and 0.22, for BFR0, BFR10, BFR20, and BFR40 (Figure 3).

### Progressive Loading and Fatigue Tests

A significant main “time” effect was observed for all these variables (p < 0.001), but no significant “group × time” interactions were detected (Table 3). All groups significantly improved in all strengthrelated parameters (i.e., 1RM, AV, AV ≥ 1, AV < 1, and MNR). ES values showed an inverted U-shaped relationship between performance gains and VL thresholds for all these variables (Figure 3).

**TABLE 3:** Changes in strength and jump performance following full-squat blood flow restricted training programs with different velocity loss thresholds.

	BFR0			BFR10			BFR20			BFR40			p-value time effect	p-value group × time

	Pre	Post	Δ (%)	Pre	Post	Δ (%)	Pre	Post	Δ (%)	Pre	Post	Δ (%)		
**1RM**(kg)	102.9 ± 20.9	115.1 ± 22.8^***^	11.8	101.8 ± 19.8	117.5 ± 15.5^***^	15.4	107.3 ± 25.6	125.6 ± 27.7^***^	17.1	106.0 ± 23.1	115.9 ± 24.6^***^	9.3	< 0.001	0.08
**AV**(m · s^−1^)	0.96 ± 0.09	1.08 ± 0.13^***^	12.5	0.94 ± 0.09	1.08 ± 0.13^***^	14.9	0.94 ± 0.09	1.12 ± 0.08^***^	19.1	0.94 ± 0.08	1.07 ± 0.06^***^	13.8	< 0.001	0.56
**AV < 1**(m · s^−1^)	0.67 ± 0.03	0.83 ± 0.12^***^	23.9	0.68 ± 0.03	0.86 ± 0.12^***^	26.4	0.69 ± 0.03	0.89 ± 0.11^***^	29.0	0.68 ± 0.05	0.82 ± 0.08^***^	20.6	< 0.001	0.43
**AV**≥ **1**(m · s^−1^)	1.28 ± 0.09	1.36 ± 0.14^*^	6.2	1.26 ± 0.09	1.34 ± 0.12^*^	6.3	1.26 ± 0.08	1.40 ± 0.09^***^	11.1	1.26 ± 0.08	1.35 ± 0.06^**^	7.1	< 0.001	0.51
**CMJ**(cm)	40.1 ± 7.6	41.4 ± 8.4	3.2	37.9 ± 6.1	41.3 ± 4.9^***^	9.0	41.7 ± 6.7	45.1 ± 8.3^***^	8.2	38.6 ± 4.8	40.2 ± 4.9^*^	4.1	< 0.001	0.06
**MNR**(n)	8.8 ± 2.4	16.0 ± 4.6^***^	81.8	9.4 ± 2.0	19.8 ± 4.4^***^	110.6	12.6 ± 3.4	20.0 ± 6.6^***^	58.7	9.1 ± 3.0	14.2 ± 6.3^***^	56.0	< 0.001	0.09

Data are mean ± SD; BFR0: Group that trained with a velocity loss (VL) of 0% in each set (n = 11); BFR10: Group that trained with a VL of 10% in each set (n = 11); BFR20: Group that trained with a VL of 20% in each set (n = 11); BFR40: Group that trained with a VL of 40% in each set (n = 13); **Δ:** percentage of change from pre- to post-training; 1RM: one-repetition maximum; AV: average mean propulsive velocity (MPV) attained against absolute loads common to pre- and post-training in the progressive loading full-squat (SQ) test. AV ≥ 1: average MPV attained against absolute loads common to pre- and post-training that were moved equal or faster than 1 m · s^–1^; AV < 1: average MPV attained against absolute loads common to pre- and post-training that were moved slower than 1 m · s^–1^; CMJ: countermovement jump height; MNR: maximal number of repetitions performed in the fatigue test. Intragroup significant differences from Pre- to Post-protocol:

* p ≤ 0.05,

** p ≤ 0.01,

*** p ≤ 0.001.

**FIG. 4:**
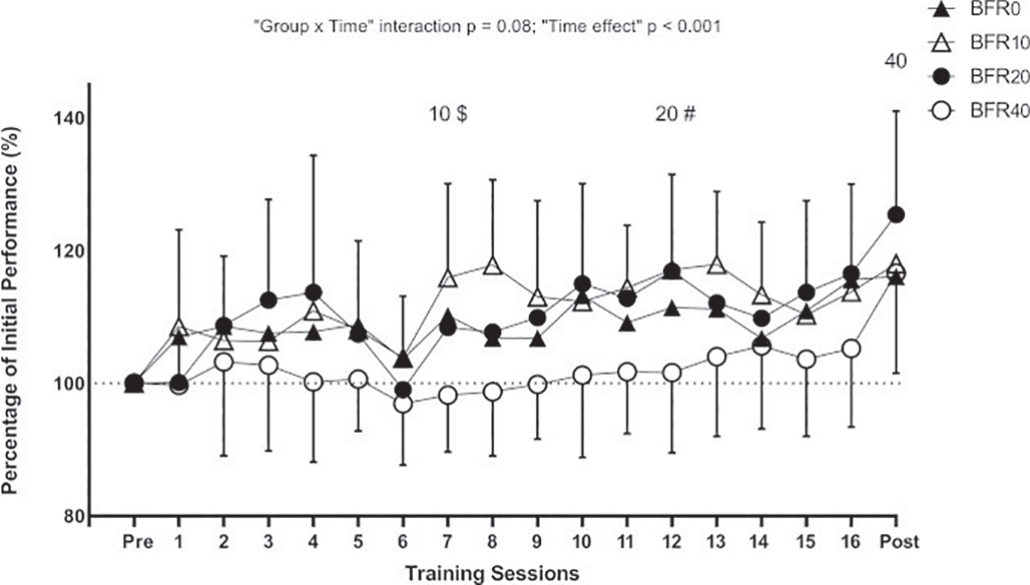
Evolution of performance in the squat exercise in each training session expressed as a percentage of the initial pre-training level for each experimental group. 10, 20, and 40 indicate the session from which the respective group attained significant improvements (p < 0.05) in strength performance compared to their pre-training values. $ Significant inter-group differences between BFR10 and BFR40 (p < 0.05). # Significant inter-group differences between BFR20 and BFR40 (p < 0.05).

During the training programme, BFR10 and BFR20 showed greater performance with the 50% 1RM load than BFR40 in sessions 7 and 9, respectively (Figure 4).

## DISCUSSION

To the best of our knowledge, this is a pioneer study examining the long-term effects of BFR-RE while controlling the level of effort through the VL. The key findings of this investigation were as follows: during the SQ exercise in a BFR context a) the higher the VL, the higher was the muscle hypertrophy; b) only the group training with a moderate VL threshold (i.e., BFR20) significantly improved in force production at various time intervals (from RFD_0–50_ to RFD_0–400_); and c) the inverted U-shaped relationships between performance gains and VL thresholds indicate that low-to-moderate VL thresholds (i.e., BFR10 and BFR20) maximized strength and jump performance improvements.

Regarding muscle size adaptations, higher VL thresholds with moderate loads and BFR led to greater muscle hypertrophy. Similar trends have been reported in studies using VL in a free-flow (FF) context [15–18], suggesting that high VL thresholds are necessary to maximize hypertrophy. Higher VL thresholds induce greater metabolic stress [12], muscle damage, and hormonal responses [27, 28]. Interestingly, higher VL thresholds induced higher muscle hypertrophy mainly in the most proximal part of the vastus lateralis (i.e., ACSA_75_ and ACSA_57.5_), without differences between groups in the most distal one. It is likely that the greatest effects are produced in the regions closest to the cuff placement. However, studies have shown mixed results depending on cuff application and protocol design. Kacin and Strazar [29] reported a trend for the increases in quadriceps ACSA to decrease at the level of cuff application. However, Sieljacks et al. [7] observed similar increases in average ACSA of quadriceps muscle at the proximal (60% and 50% of the femur length) and distal part of the cuffs (average 40%, 32.5%, and 25% of the femur length). Further research is necessary to clarify how cuff location influences regional hypertrophy.

It is well established that achieving strength gains does not require induction of high fatigue levels within the set. Our results are in line with previous research conducted in an FF context [16, 30], demonstrating that low-to-moderate VL thresholds (i.e., 10% and 20%) maximize SQ strength gains in different loading conditions (i.e., 1RM, AV, AV ≥ 1, AV < 1, and MNR). Limited studies have investigated the effects of varying levels of effort within the BFR context. Sieljacks et al. [7] compared a failure group vs. a non-failure group (which performed 75% of repetitions of the failure group) over 8 weeks (22 sessions at 25% 1RM of unilateral knee extension exercise). These authors reported similar enhancements in maximal dynamic strength, measured via 3RM (failure: 6.8% vs. non-failure: 8.9%) and strength-endurance capacity, assessed as the volume lifted with 25% 1RM until failure (failure: 13.9% vs. non-failure: 18.6%). These findings suggest that lower fatigue levels from nonfailure protocols may optimize strength gains in BFR contexts. Remarkably, our study evaluated the full spectrum of VL thresholds to determine optimal fatigue levels for enhancing various strength-derived outcomes, providing novel insights that may help refine BFR training prescription for maximizing performance with minimal fatigue. In accordance with the results already reported in FF contexts, it is demonstrated that it is not necessary to reach extreme levels of fatigue within the set to maximize gains, even when training in BFR conditions.

Regarding jump performance, an inverted U-shaped relationship was also observed between jump performance gains and VL within the set, suggesting that high VL thresholds are not optimal for improving jump height. This is also in line with previous VL literature conducted in FF contexts [15, 16, 31]. A recent review [32] highlighted that conventional light-load and low-velocity BFR protocols have a negligible impact on jump performance. In this regard, only the group that optimized jump performance gains (i.e., BFR20) elicited significant improvements in force production at different time intervals. Maximizing jump performance and RFD improvements might require lifting with maximal velocity and few repetitions, which is far from the high-fatigue BFR protocols.

The results of the present study contribute to improving the knowledge about design resistance training incorporating BFR. To optimize strength performance and force production related to the time available, practitioners should prioritize low-to-moderate VL thresholds, such as 10–20%, within sets. This approach also minimizes unnecessary fatigue and excessive volume load. Implementing higher VL thresholds (e.g., 40%) may be necessary for individuals aiming to maximize muscle hypertrophy. However, this should be carefully balanced against the risk of excessive fatigue, which may diminish strength performance-related adaptations. Tailoring VL thresholds based on specific goals ensures effective and efficient BFR training strategies, highlighting the importance of individualized programming in athletic and rehabilitation contexts.

When interpreting our findings, it is important to remark on some limitations. First, protein intake and daily diet were not monitored during the intervention, which might have affected the resulting muscle adaptations. Secondly, these results should be applied only to the same population and under identical BFR and training load conditions. Altering these factors might produce different outcomes. In view of the CV values, the extrapolations of RFD results should be treated with caution. Moreover, small variations in probe placement, pressure, or angle can lead to inconsistent CSA measurements when using ultrasonography. However, in our study, these aspects were carefully controlled to ensure measurement consistency, as reflected by the low variation (CV = 3.8%). Lastly, the study exclusively examined a multi-joint exercise (i.e., SQ). These findings may not apply to single-joint exercise scenarios or upper-body exercises.

## CONCLUSIONS

In summary, in a BFR-RE context, low-to-moderate VL thresholds (i.e., BFR10 and BFR20) were optimal for maximizing strength improvements across a spectrum from light to heavy loads. Importantly, inducing higher fatigue levels within the set was unnecessary to obtain additional gains in strength and jump performance. Additionally, a moderate level of effort (i.e., BFR20) was particularly effective in promoting RFD improvements. However, to maximize muscle hypertrophy, high VL thresholds were required (i.e., BFR40).
